# Evaluating patient acceptability and bowel preparation efficacy of sodium picosulfate‐magnesium citrate for colonoscopy

**DOI:** 10.1002/deo2.59

**Published:** 2021-09-28

**Authors:** Masaaki Shimada, Noboru Hirashima, Hiroaki Iwase, Masashi Saito, Hisashi Kondo, Noboru Urata, Satoshi Unita, Takashi Kondo, Daiki Tanaka, Takuya Tsunekawa, Sumie Nakamura, Miho Nishikura, Kaori Miyazawa, Kiyoko Fukuhara, Mitsuhiro Fujishiro

**Affiliations:** ^1^ Department of Gastroenterology National Hospital Organization, Nagoya Medical Center Aichi Japan; ^2^ Department of Nursing National Hospital Organization, Nagoya Medical Center Aichi Japan; ^3^ Department of Gastroenterology Graduate School of Medicine, The University of Tokyo Tokyo Japan; ^4^ Department of Gastroenterology & Hepatology Nagoya University Graduate School of Medicine Aichi Japan

**Keywords:** Ottawa bowel preparation scale, patient acceptability, sodium picosulfate‐magnesium citrate

## Abstract

**Objective:**

To evaluate patient acceptability and bowel preparation efficacy of sodium picosulfate‐magnesium citrate (PICOPREP) for colonoscopy.

**Methods:**

A questionnaire survey regarding the patient acceptability of bowel preparation agent PICOPREP was administered to 54 patients, and its efficacy was evaluated using the Ottawa Bowel Preparation Scale (OBPS).

**Results:**

Eighteen (33.3%) participants reported that PICOPREP is very easy to drink, 30 (55.5%) easy, four (7.4%) acceptable, one (1.9%) difficult, and one (1.9%) very difficult. The flavor was very good as reported by eight (14.8%) participants, good by 25 (46.3%), neutral by 20 (37.0%), bad by one (1.9%), and very bad by none. The number of patients who requested PICOPREP was 42 (77.7%), indicating its high acceptability. Evaluation of the OBPS score showed that the rectosigmoid colon had significantly better polyethylene glycol (PEG) scores than PICOPREP, but the entire colon did not show a significant difference between PICOPREP and PEG scores (1.09 ± 0.65 vs. 1.17 ± 0.76, *p* = 0.632 in the right colon; 0.48 ± 0.52 vs. 0.72 ± 0.66, *p* = 0.079 in the mid colon, 0.93 ± 0.49 vs. 0.63 ± 0.52, *p* = 0.012 in the rectosigmoid colon, and 3.28 ± 1.70 vs. 3.20 ± 1.90, *p* = 0.836 in the entire colon).

**Conclusion:**

PICOPREP is considered as one of the important options due to its good patient acceptability and high efficacy similar to PEG.

## INTRODUCTION

The incidence of colorectal cancer is increasing in Japan, with a high number of deaths in both men and women; however, it is widely known to be curable if detected and treated at an early stage.^1^ However, colorectal cancer has no subjective symptoms at an early stage. Therefore, colonoscopy is recommended for screening and early detection.^2^ Still, the current colonoscopy consultation rate remains insufficient. It is expected that if the rate of colonoscopy is improved by selecting a highly patient‐acceptable bowel preparation agent, the number of deaths from colorectal cancer will significantly reduce.

Solutions containing polyethylene glycol (PEG) and magnesium citrate preparations have been conventionally used as standard bowel preparations. However, PEG preparations have an unacceptable taste or require a large volume; thus, patient satisfaction is low. Therefore, it is considered as one of the factors that patients refuse to undergo colonoscopy.^3^, ^4^, ^5^, ^6^

During colonoscopy, the balance between the cleansing effect of the bowel preparation and the patient's acceptability should be balanced, and thus, the bowel preparation agent should be selected according to the purpose of the examination, the condition, or the patient's preference.

Sodium picosulfate–magnesium citrate (PICOPREP; Nippon‐Chemiphar Ltd, Tokyo, Japan) became commercially available in Japan in 2016 and has two different actions: stimulant laxative picosulfate sodium hydrate and salt laxative magnesium citrate. Its flavor is orange.^7^

The efficacy rate calculated based on the general colon cleansing, the primary efficacy endpoint in the Japanese phase III study (J‐CLEAR study), was 97.7% in the PICOPREP divided‐dose group (once the day before and once on the day), 92.0% in the day before the group (twice the day before), and 95.3% in the control group (isotonic PEG; Niflec; EA Pharma Ltd, Tokyo, Japan).^8^ These results revealed the non‐inferiority of the PICOPREP group to the control group. According to the results of the patient questionnaire, all factors, that is, the ease of drinking, overall impression, taste, and amount acceptability, were significantly better in the PICOPREP group than in the control group. The incidence of adverse events was 12.7% in the PICOPREP divided‐dose group, 15.2% in the PICOPREP the day before the group, and 15.4% in the control group, indicating that all groups had similar results.

PICOPREP considers patient acceptability by allowing them to choose a small volume of the agent (150 ml × 2 times) and a transparent drink to be taken after taking the agent according to their tastes. In addition, it is a bowel preparation that can be taken at home as per the patient's preference. These facts supported that PICOPREP considering patient acceptability will become more important as an option for bowel preparation in the future. The divided‐dose regimen of PICOPREP has been shown to be effective, safe, and acceptable.^9^, ^10^ Therefore, the method is adopted at our hospital.

In this exploratory study, we report on the patient's acceptability using the PICOPREP questionnaire and the efficacy of bowel preparation at our hospital.

## MATERIALS AND METHOD

### Patients

**FIGURE 1:**
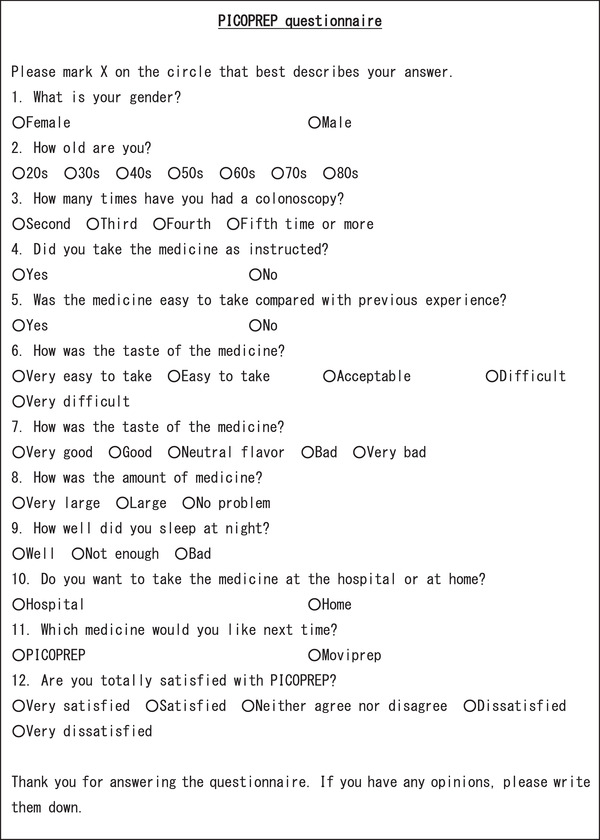
The questionnaire for sodium picosulfate‐magnesium citrate (PICOPREP)

This study was conducted at the Department of Gastroenterology, Nagoya Medical Center, between December 2017 and June 2019. All consecutive patients who experienced taking the preparation with PEG solution containing ascorbic acid (Moviprep; EA Pharma Ltd, each liter contained 100.0 g of macrogol 4000, 7.5 g of sodium sulfate, 2.7 g of sodium chloride, 1.0 g of potassium chloride, 4.7 g of ascorbic acid, 5.9 g of sodium ascorbate, and lemon flavoring) were scheduled for a colonoscopy for screening, diagnostic evaluation, or surveillance of ulcerative colitis (UC) by two gastroenterologists who specialize mainly in inflammatory bowel disease (Masaaki Shimada and Hiroaki Iwase), and they completed a questionnaire on the patient's acceptability of PICOPREP which was handed out by a nurse after colonoscopy. The questionnaire's contents included sex, age, ease of drinking, taste, volume, choice of place to take pretreatment drug (at a hospital or home), and preparation agent for the next procedure compared with previous PEG experience (Figure 1). In addition, alcohol consumption (daily alcohol consumption exceeding 20 g in women or 30 g in men), smoking status (current smoker, former smoker, and never smoker), history of diabetes mellitus, hypertension, or abdominal surgery, and the presence or absence of constipation (two or fewer defecations per week) were obtained from medical records. The sample size was determined based on the number of patients in our hospital during the study period. The questionnaire was administered to 67 outpatients, and 13 patients were excluded because they did not fill out the questionnaire or did not provide an appropriate answer. Thus, a total of 54 patients were analyzed.

### Picoprep instruction

The patient was advised by the medical staff about the proper use of bowel preparation methods. The patient commenced a minimal residue diet the day before colonoscopy and received a handout listing of food and fluids to avoid. The PICOPREP content of each sachet (sodium picosulfate 10 mg; magnesium oxide 3.5 g, and citric acid 12 g) was dissolved in 150 ml of water at a time and was taken twice the night before (5 p.m.–9 p.m.) and the morning of the day of the colonoscopy. Afterward, 250 ml of clear fluids should be drunk at least five times, and after the second dose, 250 ml of clear fluids should be drunk at least three times. Four sennoside A B calcium tablets and one new lecicarbon suppository were added the night before colonoscopy. The total amount of water intake was set at 2500 ml or more.

### Quality of bowel preparation

The Ottawa Bowel Preparation Scale (OBPS) was evaluated by one gastroenterologist (Masaaki Shimada) to assess the quality of bowel preparation (Table 1).^11^ In the OBPS, bowel cleanliness and fluid volume were assessed separately. The right, mid, and rectosigmoid colons were individually assessed for bowel cleanliness using a scale from 0 to 4. In each segment, a score of ≥3 is evaluated as inadequate bowel preparation because the mucosa could not be clearly observed despite washing and suctioning due to residual defecation. In addition to the segment score, fluid scores reflecting the fluid volume throughout the bowel were assessed using a scale from 0 to 2. A score of 0 indicates a small fluid volume, 1 indicates a moderate volume, and 2 indicates a large volume. The total bowel preparation score was calculated by adding the three major segment scores and the fluid score. The total bowel preparation score ranged from 0 to 14, and a score of ≥8 was defined as inadequate bowel preparation.

**TABLE 1:** Ottawa bowel preparation scale

Preparation quality	Score
Individual evaluation of the right, mid, and rectosigmoid colon	
No liquid	0
Minimal liquid, no suction needed	1
Suction needed	2
Suction and wash needed	3
Solid stool, not washable	4
Evaluation of the entire colon	
Overall fluid quantity	0–2

*Note*: Total Ottawa bowel preparation scale (0–14) is obtained by adding the scores for individual evaluation of the right, mid, and rectosigmoid colon with the score of overall fluid in the entire colon.

### Statistical analyses

All data were expressed as mean ± standard deviation. Intergroup differences were analyzed using a paired *t*‐test, the Mann–Whitney *U* test, and Fisher's exact test. Values of *p* < 0.05 were considered to indicate statistical significance.

This study was approved by the Ethics Review Board of Nagoya Medical Center.

## RESULTS

### Patients’ characteristics

A total of 54 patients were enrolled in this study between December 2017 and June 2019. All patients underwent total colonoscopy. All responded to the questionnaire on the acceptability of PICOPREP. The mean age of patients was 53.9 ± 17.4 years, and 29 (53.7%) of them were men. Colonoscopy was indicated for screening in five patients, fecal occult blood positive in five, UC in 33, and colorectal polyps in 11, and the rate of UC was high (61.1%) (Table 2). Therefore, the background was compared between the UC and non‐UC groups. The UC group was significantly younger and had more colonoscopies than the non‐UC group. No significant differences in other factors were found between the two groups (Table 3).

**TABLE 2:** Baseline patient characteristics

**Age, years (±SD)**	**53.9 ± 17.4**
Sex, *n* (male/female)	29/25
Smoker, *n* (%)	7 (13.0)
Drinker, *n* (%)	7 (13.0)
Lifetime experiences of colonoscopy, *n* (%)	
1	0 (0)
2	6 (11.1)
>2	48 (88.9)
Previous colonoscopy (±SD)	5.8 ± 5.0
Indication for colonoscopy, *n* (%)	
Screening	5 (9.3)
Positive for fecal occult blood	5 (9.3)
Ulcerative colitis	33 (61.1)
Surveillance of colorectal polyps	11 (20.4)
Patient medical history, *n* (%)	
Hypertension	8 (14.8)
Diabetes mellitus	4 (7.4)
Previous abdominal surgery, *n* (%)	4 (7.4)
Constipation, *n* (%)	1 (1.9)

**TABLE 3:** Baseline patient characteristics

	**UC (*n* = 33)**	**Non‐UC (*n* = 21)**	***p*‐value**
Age, years (±SD)	53.9 ± 17.4	64.1 ± 13.0	<0.001
Sex, *n* (male/female)	17/16	12/9	0.686
Smoker, *n* (%)	3 (9.1)	4 (19.0)	0.288
Drinker, *n* (%)	3 (9.1)	4 (19.0)	0.288
Lifetime experiences of colonoscopy, *n* (%)			0.013
1	0 (0.0)	0 (0.0)	
2	2 (6.1)	8 (38.1)	
>2	31 (93.9)	13 (61.9)	
Previous colonoscopy (±SD)	7.5 ± 5.4	3.1 ± 2.7	<0.001
Patient medical history, *n* (%)			
Hypertension	4 (12.1)	4 (19.0)	0.485
Diabetes mellitus	3 (9.1)	1 (4.8)	0.554
Previous abdominal surgery, *n* (%)	2 (6.1)	2 (9.5)	0.636
Constipation, *n* (%)	0 (0.0)	1 (4.8)	0.206

Abbreviation: UC, Ulcerative colitis.

### Patients’ acceptability

According to the questionnaire on PICOPREP acceptability (Figure 1), the ease of drinking PICOPREP was very easy in 18 (33.3%), easy in 30 (55.5%), acceptable in four (7.4%), difficult in one (1.9%), and very difficult in one (1.9%) patient. The taste was very good in eight (14.8%) patients, good in 25 (46.3%), neutral flavor in 20 (37.0%), bad in one (1.9%), and very bad in none (0%) (Table 4). The volume was no problem in 51 (94.4%) patients, high in two (3.7%), and very high in one (1.9%) patient. Eighteen patients (33.3%) experienced sleep disorders as a minor adverse event. The choice for the place, at home or hospital, to take PICOPREP in the future were 41 (75.9%) at home, 11 (20.4%) at the hospital, and two (3.7%) at either. The reason was that out of 41 patients who preferred to stay at home, 30 patients (73.2%) had free access to the restroom, 16 patients (39.0%) could use their time effectively, and 15 patients (36.6%) felt relaxed (including duplicate answers). On the other hand, five out of 11 patients (45.5%) had problems with defecation from home to the hospital, so they would rather take PICOPREP at the hospital. The next acceptable preparation agent selection was PICOPREP in 42 (77.7%), PEG in 11 (20.4%), and whichever in one (1.9%) patient. Therefore, most patients accepted the PICOPREP preparation.

**TABLE 4:** Convenience and flavor of the cleansing agent

**Convenience**	***n* (%)**	**Flavor**	***n* (%)**
Very easy	18 (33.3)	Very good	8 (14.8)
Easy	30 (55.5)	Good	25 (46.3)
Acceptable	4 (7.4)	Neutral flavour	20 (37.0)
Difficult	1 (1.9)	Bad	1 (1.9)
Very difficult	1 (1.9)	Very bad	0 (0.0)

### Bowel preparation

The total amount of water intake was 3232.6 ± 754.6 ml in all patients, 3275.5 ± 833.5 ml in the UC group, and 3165.2 ± 624.3 ml in the non‐UC group (*p* = 0.901). Total OBPS scores of PICOPREP and PEG were 3.28 ± 1.70 (range, 0–9) versus 3.20 ± 1.90 (range, 0–8), *p* = 0.836. Breakdowns of OBPS scores into scores of each colonic segment and fluid volume were 1.09 ± 0.65 versus 1.17 ± 0.76, *p* = 0.632; 0.48 ± 0.52 versus 0.72 ± 0.66, *p* = 0.079; 0.93 ± 0.49 versus 0.63 ± 0.52, *p* = 0.012; and 0.78 ± 0.26 versus 0.69 ± 0.26, *p* = 0.301, in the right colon, the mid colon, the rectosigmoid colon, and fluid volume, respectively. The rectosigmoid colon scores were significantly better in PEG than in PICOPREP, whereas the total OBPS scores did not show a significant difference (Table 5). Bowel preparation was inadequate in the non‐UC group, having one (1.9%) with PICOPREP, and two (3.7%) with PEG, based on the colonoscopy results. Analyzing poor bowel preparation in each colonic segment, we observed two (3.7%) occurring in the right colon, none (0%) in the mid colon, one (1.9%) in the rectosigmoid colon in PICOPREP, six (11.1%) in the right colon, one (1.9%) in the mid colon, and none (0%) in the rectosigmoid colon in PEG. Furthermore, there were no significant differences between PICOPREP and PEG in the total OBPS score in both groups, but in PICOPREP, the OBPS score of the UC group was significantly superior to that of the non‐UC group in the rectosigmoid colon (0.75 ± 0.42 vs. 1.20 ± 0.57, *p* = 0.012; Tables 6 and 7).

**TABLE 5:** Ottawa Bowel Preparation Scale (OBPS) score

	**PICOPREP**	**PEG**	***p*‐value**
Entire colon	3.28 ± 1.70	3.20 ± 1.90	0.836
Right colon	1.09 ± 0.65	1.17 ± 0.76	0.632
Mid colon	0.48 ± 0.52	0.72 ± 0.66	0.079
Rectosigmoid colon	0.93 ± 0.49	0.63 ± 0.52	0.012

Abbreviations: PEG, solutions of polyethylene glycol; PICOPREP, sodium picosulfate‐magnesium citrate.

**TABLE 6:** Ottawa Bowel Preparation Scale (OBPS) score (sodium picosulfate‐magnesium citrate [PICOPREP])

	**UC (*n* = 33)**	**Non‐UC (*n* = 21)**	***p*‐value**
Entire colon	2.94 ± 1.55	3.81 ± 1.91	0.207
Right colon	1.09 ± 0.68	1.10 ± 0.61	0.903
Mid colon	0.39 ± 0.51	0.62 ± 0.51	0.181
Rectosigmoid colon	0.75 ± 0.42	1.20 ± 0.57	0.012

**TABLE 7:** Ottawa Bowel Preparation Scale (OBPS) score (solutions of polyethylene glycol [PEG])

	UC (*n* = 33)	Non‐UC (*n* = 21)	*p‐*value
Entire colon	2.97 ± 1.89	3.57 ± 1.92	0.388
Right colon	1.09 ± 0.74	1.29 ± 0.79	0.511
Mid colon	0.76 ± 0.71	0.67 ± 0.56	0.885
Rectosigmoid colon	0.52 ± 0.42	0.81 ± 0.61	0.213

## DISCUSSION

In order to reduce the burden on patients with bowel preparation for colonoscopy, taking a bowel preparation agent at home is considered as one of the important methods. This method benefits both patients and medical staff. From the patient's point of view, in addition to being able to take bowel preparation agents at home at the patient's own pace, they can use the restroom individually without consideration of others, which can help reduce mental burden. Furthermore, since the preparation has been completed at home, a colonoscopy can be performed immediately after visiting the hospital. From the perspective of hospital facilities, if the number of colonoscopies performed is high, the number of restrooms may be insufficient. However, if the preparation agent is taken at home, the number of restrooms will be less of a problem.

Coronavirus disease 2019 (COVID‐19; a new type of coronavirus infection), which occurred in early December 2019 and is prevalent in 2020, is isolated from airway secretions and feces.^12^ Therefore, the use of the same restroom by multiple people in a hospital during colonoscopy preparation is feared to might increase the risk of COVID‐19 infection. In that respect, bowel preparation using PICOPREP is considered to be beneficial for patients and medical staff because it completes preparation at home, leading to a significant reduction in COVID‐19 infection risk.

In addition to PICOPREP's domestic phase III study (J‐CLEAR study),^8^ a meta‐analysis of 25 randomized controlled trials comparing PICOPREP with PEG (Moviprep) showed non‐inferiority by bowel cleansing effect, polyp detection rate, and adenoma detection rate.^13^ PICOPREP requires a small volume of bowel preparation agent solution (150 ml × 2 times), and patients can choose to drink clear fluids after taking the agent solution according to their tastes, resulting in high patient acceptability.^14^, ^15^, ^16^ Furthermore, patients can select from two dosing schedules according to the facility and the patient's situation. However, special consideration should be made for patients with renal failure and congestive heart failure, and elderly people.^17^

According to the consensus statement and guidelines compiled by the American and European academic societies, the effectiveness of PICOPREP is evaluated to be almost the same as that of conventional PEG.^18^, ^19^ Moreover, in a multicenter observational study conducted in France on the best colonoscopy preparation agent for inflammatory bowel disease, both agents PICOPREP and PEG‐2L were equally safe and were reported to have significantly better efficacy and patient acceptability than PEG‐4L.^20^ In this study, UC was observed in 33 patients (61.1%), a well‐known cause of unknown chronic diseases. Therefore, a stable remission period should be considered for long‐term treatment. In addition, colonoscopy is performed regularly because of the risk of colorectal cancer in UC.^21^, ^22^, ^23^ Therefore, a bowel preparation agent with high patient acceptability should be selected in order to evaluate the mucosal condition or follow‐up UC.^24^, ^25^ In this respect, PICOPREP is highly acceptable for UC, and its bowel preparation level is comparable to that of conventional PEG preparations. In this study, the quality of bowel preparation in the UC group was significantly superior in the rectosigmoid colon compared to the non‐UC group. Therefore, it is estimated as a bowel preparation agent for patients with UC.

This study has several limitations. First, making a detailed evaluation of the degree of bowel preparation is difficult based on the patient's background because this was an exploratory study with a small number of patients in a single facility. In particular, the disease is biased toward UC. Second, PICOPREP and PEG were not evaluated at the same time. Therefore, it is considered necessary to compare PICOPREP with conventional PEG in order to clarify patient acceptability and bowel preparation effectiveness for PICOPREP in future studies. Finally, no patient survey was conducted on the acceptability of PEG.

In conclusion, PICOPREP has a good balance between the cleansing effect of bowel preparation and patient acceptability during colonoscopy. Therefore, it is considered as one of the preparation options for colonoscopy. It is expected to be useful for lowering the mortality rate of colorectal cancer by improving the consultation rate of colonoscopy and the surveillance of UC.

## CONFLICT OF INTEREST

The authors declare that they have no conflict of interest.

## FUNDING INFORMATION

None
